# The carrier rate and mutation spectrum of genes associated with hearing loss in South China hearing female population of childbearing age

**DOI:** 10.1186/1471-2350-14-57

**Published:** 2013-05-29

**Authors:** Aihua Yin, Chang Liu, Yan Zhang, Jing Wu, Mingqin Mai, Hongke Ding, Jiexia Yang, Xiaozhuang Zhang

**Affiliations:** 1 Prenatal Diagnosis Centre, Guangdong Women and Children Hospital, Guangzhou, Guangdong 510010, China; 2 Maternal and Children Metabolic-Genetic Key Laboratory, Guangdong Women and Children Hospital, Guangzhou, Guangdong 510010, China

**Keywords:** Hearing loss, Carrier rate, Mutation spectrum

## Abstract

**Background:**

Given that hearing loss occurs in 1 to 3 of 1,000 live births and approximately 90 to 95 percent of them are born into hearing families, it is of importance and necessity to get better understanding about the carrier rate and mutation spectrum of genes associated with hearing impairment in the general population.

**Methods:**

7,263 unrelated women of childbearing age with normal hearing and without family history of hearing loss were tested with allele-specific PCR-based universal array. Further genetic testing were provided to the spouses of the screened carriers. For those couples at risk, multiple choices were provided, including prenatal diagnosis.

**Results:**

Among the 7,263 normal hearing participants, 303 subjects carried pathogenic mutations included in the screening chip, which made the carrier rate 4.17%. Of the 303 screened carriers, 282 harbored heterozygous mutated genes associated with autosomal recessive hearing loss, and 95 spouses took further genetic tests. 8 out of the 9 couples harbored deafness-causing mutations in the same gene received prenatal diagnosis.

**Conclusions:**

Given that nearly 90 to 95 percent of deaf and hard-of-hearing babies are born into hearing families, better understanding about the carrier rate and mutation spectrum of genes associated with hearing impairment in the female population of childbearing age may be of importance in carrier screening and genetic counseling.

## Background

In China, approximately 30,000 infants are born with congenital hearing loss per 20 million live births every year [1,2]. Moreover, a similar number are affected with hearing impairment before adulthood. Children with hearing loss may confront with a series of obstacles, given that spoken language is the predominant medium of interpersonal communication and social interaction [3]. Adequate auditory stimulation and sufficient language exposure in early childhood are critical in their subsequent linguistic acquisition, cognitive development, as well as in psychosocial functioning [3-6]. In recognition of the ever-increasing and tremendous burden of hearing impairment globally, the World Health Assembly (WHA) passed a resolution on the prevention and control of major causes of hearing loss [3,7]. As estimated, more than 50 percent of prelingual hearing loss are attributable to genetic factors [8]. Given that approximately 90 to 95 percent of deaf and hard-of-hearing babies are born into hearing families [9], better understanding about the carrier rate and mutation spectrum of genes associated with hearing loss in the female population of childbearing age may be of vital importance in carrier screening, genetic testing, as well as in providing accurate counseling.

Despite a great number of mutations in many genes may contribute to hearing loss, the patterns of high frequency mutations in certain populations and their population-wide distributions make it meaningful to initially select hotspot mutations for carrier screening [10,11]. A microarray developed by CapitalBio (CapitalBio, Beijing, China) was designed to detect nine hotspot mutations in Chinese population, based on large-scale epidemiological studies across 28 provinces and municipalities [12-16], indicating that the majority (>80%) of hereditary hearing loss in Chinese patients were related to those nine hotspot mutations. However, the carrier rate and mutation spectrum of deafness- associated genes in the general population was still under the veil. The parallelism offered by the microarray platform makes it suited for genotyping of genetically heterogeneous condition of hearing loss [17-19], especially in the carrier screening practice.

## Methods

### Subjects and DNA samples

A total of 7,263 unrelated women of childbearing age with normal hearing and without family history of hearing loss were enrolled in the study under an institutional review board-approved protocol of informed consent at the Guangdong Women and Children Hospital Institutional Review Board and Ethics Committee of Guangzhou Medical College, China. The study cohort was recruited from women came for (pre-)pregnancy examination at the Clinic of Guangdong Women and Children Hospital, China. The majority of the participants were from Guangdong Province, and a small portion of them were from Hunan, Guangxi and Jiangxi Provinces. The study cohort was of a significant characteristic of South China origins. A comprehensive history and physical examination record for each participating subjects was obtained, including clinical history, infaust gravidity, infection, aminoglycoside antibiotics exposure, as well as genetic factors related to the hearing loss. Blood samples were obtained from all participants and genomic DNA was isolated from the whole blood by standard procedures using Fuji DNA Blood Minikit.

### Carrier screening

Allele-specific PCR-based universal array was applied to screen mutation carriers of deafness-associated genes in the study cohort [10]. The Detection Array Kit (Registration Certificate for Medical Device No. 0903084, by Chinese State Food and Drug Administration) was purchased from CapitalBio Corporation (Beijing, China). According to the manufacturer’ protocol, asymmetric PCR was used to obtain sufficient single-strand DNA for hybridization. After heat denaturation procedure, the hybridization mixture was applied to the array. The slide was incubated at 50°C for 1 hour, and washed twice at 42°C in 0.3% SSC/0.1% SDS and in 0.06% SSC. Finally, the chip was imaged with a LuxScan™ 10 K-B Microarray Scanner (CapitalBio, Beijing, China). The statistical analysis was performed using SPSS 17.0 (SPSS Inc., Chicago, USA).

### Genetic counseling and prenatal diagnosis

For the mutation carriers of genes associated with hearing loss, elaborate genetic counseling were provided, concerning the nature of the disease, the implications of being carriers and the reproductive choices [20,21]. Further genetic testing, both allele-specific PCR-based universal array detection and DNA sequence analysis, were provided to the spouses of screened carriers.

As for those couples at risk and may need prenatal diagnosis, detailed information was offered, regarding to the fetal DNA sampling procedure, the risk of fetal mortality, the chance of misdiagnosis, and the mortality and morbidity of an abortion in case of affected fetus [22]. Fetal DNA for analysis can be obtained from either chorionic villi, amniotic fluid, or fetal blood.

## Results

### The carrier rate and mutation spectrum of deafness-associated genes

Among the 7,263 normal hearing participants enrolled in this study, 303 subjects carried pathogenic mutations included in the screening chip, which made the carrier rate 4.17% in the population under investigate. As shown in Table 1, a total of 164 participating subjects carried one type of the four common deafness-causing mutations in GJB2 gene in the heterozygous state, and the carrier rate was 2.26% in the population. Frame-shift c. 235 del C was the most prevalent mutation in the Chinese hearing population with a carrier rate of 1.76%. Followed by c. 299_300 del AT variation, the carrier rate of which was 0.38%. Frame-shift c. 176_191 del 16 bp accounted for 0.10% in group. Pathogenic mutation c. 35 del G accounted for 0.01% in the population under study. Moreover, 109 subjects were found carrying one type of the two confirmed pathogenic mutations in SLC26A4 gene in the heterozygous state, which accounted for 1.50% in the Chinese hearing population under study. Aberrant splice-site alteration c. 919–2 A > G accounted for 1.24% in the group, and the carrier rate of c. 2168 A > G mutation was 0.26%. Furthermore, 9 subjects were found carrying pathogenic mutation c. 538 C > T in GJB3 gene in the heterozygous state, which accounted for 0.12% in the Chinese hearing population under investigate. In addition, 21 participants were detected to be homoplasmy mutation carriers of mitochondrial DNA 12S rRNA gene, accounting for 0.29% of the group. The carrier rate of m. 1555 A > G was 0.25% in the population under investigate, and m. 1494 C > T variation accounted for 0.04% of the group. Since mitochondrial DNA 12S rRNA gene mutations was important mechanism of genetic susceptibility to aminoglycoside ototoxicity, screened carriers were provided with detailed medication guide.

**Table 1:** The Carrier rate and mutation spectrum of deafness-associated genes in 7,263 hearing subjects

**Gene**	**Nucleotide change**	**Carrier number**	**Carrier rate (%)**
GJB2 gene	c. 35 del G	1	0.01%
	c. 176_191 del 16 bp	7	0.10%
	c. 235 del C	128	1.76%
	c. 299_300 del AT	28	0.38%
GJB3 gene	c. 538 C > T	9	0.12%
SLC26A4 gene	c. 2168 A > G	19	0.26%
	c. 919–2 A > G	90	1.24%
mtDNA 12S rRNA gene	m. 1494 C > T	3	0.04%
	m. 1555 A > G	18	0.25%

### Genetic test for the spouses of screened carriers

Of the 303 screened carriers, 282 were found out to be carrying heterozygous mutated genes associated with autosomal recessive hearing loss. After the genetic counseling, with regard to the nature of the disease, the implications of being carriers and the reproductive choices, were provided to the carriers and their families, 95 spouses were willing to take further genetic tests, including the allele-specific PCR-based universal array to detect high frequency deafness-associated mutations in Chinese population, and DNA sequencing to analyze the mutated gene of the wife. As shown in Additional file 1: Table S1, nine couples harbored deafness-causing mutations in the same gene.

### Prenatal diagnosis and pregnancy outcomes

In the present study, eight out of the nine couples harbored deafness-causing mutations in the same gene received prenatal diagnosis (Table 2). The pregnancy outcomes were in accordance with the prenatal diagnosis results. Five fetuses were mutation carriers and one harbored neither of the deafness-causing mutations of the parents, performed normal hearing after births. One fetus carried double deafness-causing mutations and developed server hearing loss after birth. Detail information and early intervention programs were provided to his parents. The other couple with the fetus harbored double deafness-causing mutations chose to terminate the affected pregnancy. Another couple carrying heterozygous mutated genes associated with autosomal recessive hearing loss decided not to have prenatal diagnosis. Their baby was born with moderate hearing loss, and was provided with support services and early intervention programs.

**Table 2:** Prenatal diagnosis and pregnancy outcomes of couples harbored deafness-causing mutations in the same gene

**Family**	**Mutated gene**	**Genotype of the wife**	**Genotype of the husband**	**Genotype of the fetus**	**Pregnancy outcome**
F1	GJB2	Heterozygous c. 299_300 del AT	Heterozygous c. 235 del C	Heterozygous c. 235 del C	Normal hearing
F2	GJB2	Heterozygous c. 235 del C	Heterozygous c. 235 del C	Homozygous c. 235 del C	Severe hearing loss
F3	GJB2	Heterozygous c. 299_300 del AT	Heterozygous c. 235 del C	Heterozygous c. 299_300 del AT	Normal hearing
F4	GJB2	Heterozygous c. 235 del C	Heterozygous c. 235 del C	Homozygous c. 235 del C	Termination of pregnancy
F5	GJB2	Heterozygous c. 235 del C	Heterozygous c. 299_300 del AT	Wild type	Normal hearing
F6	SLC26A4	Heterozygous c. 919–2 A > G	Heterozygous c.1548 ins C	Heterozygous c. 919–2 A > G	Normal hearing
F7	GJB2	Heterozygous c. 235 del C	Heterozygous c. 512 ins AACG	Heterozygous c. 235 del C	Normal hearing
F8	GJB2	Heterozygous c. 235 del C	Heterozygous c. 109 G > A	Heterozygous c. 109 G > A	Normal hearing
F9	GJB2	Heterozygous c. 235 del C	Heterozygous c. 109 G > A	Not know (refuse prenatal diagnosis)	Moderate hearing loss

## Discussion

In the present study, a cohort of 7,263 unrelated women of childbearing age were investigated for the carrier rate of genes associated with hearing loss in the South China hearing female population. In the study cohort, 303 subjects carried pathogenic mutations associated with hearing loss, which made the carrier rate 4.17% in the population. Based on a comparatively large study population, the figure could be reliable and representative, and could be of importance in carrier screening and genetic counseling of hearing loss, the most common birth defect and the most prevalent sensorineural disorder in China.

Besides of the carrier rate, the mutation spectrum of genes associated with hearing loss in South China hearing population was also a core concern in the present study. As previous reports suggested, variants of GJB2 gene could account for the molecular etiology of 8 to 72 percents of non-syndromic hearing impairment. In the study cohort, GJB2 gene mutations accounted for 54.12% of the mutant alleles. The most common mutation in Caucasians, c. 35 del G, accounted for only 0.33% of mutant alleles in the South China hearing population. Instead, c. 235 del C accounted for 42.24% of mutant alleles in the study population. c. 235 del C was also at the highest rate in the Asian deaf populations [23]. Moreover, two pathogenic mutations c. 919–2 A > G and c. 2168 A > G in SLC26A4 gene were detected in 35.97% of mutant alleles in the study cohort, with c. 919–2 A > G being the most prevalent mutant form in SLC26A4 gene. The deafness-associated variation c. 538 C > T in GJB3 gene was considered not common in the study cohort. In addition, important causes of aminoglycoside-induced and non-syndromic hearing loss, mitochondrial DNA 12S rRNA gene m. 1555 A > G and m. 1494 C > T variations accounted for 6.93% of mutant alleles in the South China hearing population. The carrier rate of mtDNA 12S rRNA gene mutations in the study cohort was higher than that in the previous reports based on populations from other parts of China, which may be explained in phylogenetic insights [24,25].

The patterns of high frequency mutations in populations and their population-wide distributions made it meaningful to initially select hotspot mutations for carrier screening. Yet the genetic heterogeneity of hearing loss has proved a challenge for genetic testing using conventional methods. Fortunately, the parallelism offered by the microarray platform makes it ideally suited for genotyping of genetically heterogeneous conditions such as hearing loss. The allele-specific PCR-based universal array allowed the parallel analysis of nine common variations in GJB2, GJB3, SLC26A4, and mitochondrial DNA 12S rRNA genes, and could easily be expanded or modified, according to further epidemiologic survey on the carrier rate and mutation spectrum of genes associated with hearing loss under certain ethnic background.

## Conclusion

Previous researches mainly focused on molecular etiology of hearing loss, which provided valuable data of deafness-causing mutations and their prevalences. However, without the implementation of carrier screening and intervention programs, the average age at diagnosis was 1.5 to 3 years, which was well beyond the beginning of the critical interval for speech and language acquisition [26]. Diagnostic delay has a profound impact on communicative competence, as well as cognitive and psychosocial development. Considering the fact that hearing loss occurs in 1 to 3 of 1,000 live births annually and approximately 90 to 95 percent of them are born into hearing families, it is of importance and vital necessity to get better understanding about the carrier rate and mutation spectrum of genes associated with hearing impairment in the general population. It would be the first step for constructing a national database, and would then provides valuable information to health planners and policy makers in planning programs and better allocating resources. Moreover, since aminoglycoside ototoxicity was a common health problem in China and mitochondrial DNA 12S rRNA gene mutations were supposed to be important causes of aminoglycoside-induced hearing loss, screening of mutation carriers at risk for ototoxicity and providing them with proper medication guide would help decrease the incidence of aminoglycoside-induced hearing loss.

## Competing interest

The authors declare that they have no competing interests.

## Authors’ contributions

XZ Zhang, AH Yin and C Liu defined the research theme. AH Yin, C Liu, Y Zhang, J Wu, MQ Mai, HK Ding and JX Yang performed the experimental work and organized the data. AH Yin and C Liu designed experiments, interpreted data and drafted the manuscript. XZ Zhang critically reviewed the manuscript and provided concepts. All authors read and approved the final manuscript.

## Pre-publication history

The pre-publication history for this paper can be accessed here:

http://www.biomedcentral.com/1471-2350/14/57/prepub

## Supplementary Material

Additional file 1: Table S1 Genetic test results for the spouses of screened carriers. Click here for file
