# Supplementary material for: The carrier rate and mutation spectrum of genes associated with hearing loss in South China hearing female population of childbearing age
Source: BMC Med Genet. 2013 May 29;14:57. doi: 10.1186/1471-2350-14-57 (PMC3680026; doi:10.1186/1471-2350-14-57)
Supplement: Additional file 1: Table S1 — Genetic test results for the spouses of screened carriers. [file 1471-2350-14-57-S1.doc]

**Table S1** Genetic Test Results for the Spouses of Screened Carriers

| **Subject** | **Genotype of the Wife** | **Tested Gene** | **Nucleotide Change** |
| --- | --- | --- | --- |
| S1 | Heterozygous SLC26A4 gene c. 919–2 A>G | SLC26A4 | Heterozygous IVS 15+61 C>A |
| S2 | Heterozygous GJB2 gene c. 235 del C | GJB2 | Heterozygous c. 79 G>A, c. 341 A>G |
| S3 | Heterozygous SLC26A4 gene c. 2168 A>G | SLC26A4 | Heterozygous IVS 15+61 C>A |
| S4 | Heterozygous GJB2 gene c. 235 del C | GJB2 | Heterozygous c. 608 T>C |
| S5 | Heterozygous GJB2 gene c. 235 del C | GJB2 | Wild type |
| S6 | Heterozygous GJB2 gene c. 299_300 del AT | GJB2 | Wild type |
| S7 | Heterozygous GJB2 gene c. 235 del C | GJB2 | Wild type |
| S8 | Heterozygous GJB2 gene c. 235 del C | GJB2 | Heterozygous c. 79 G>A, c. 341 A>G |
| S9 | Heterozygous GJB2 gene c. 235 del C | GJB2 | Heterozygous c. 79 G>A, c. 341 A>G |
| S10 | Heterozygous c. 176_191 del 16 bp | GJB2 | Heterozygous c. 79 G>A |
| S11 | Heterozygous SLC26A4 gene c. 919–2 A>G | SLC26A4 | Wild type |
| S12 | Heterozygous SLC26A4 gene c. 919–2 A>G | SLC26A4 | Heterozygous IVS 11+47 T>C |
| S13 | Heterozygous GJB2 gene c. 299_300 del AT | GJB2 | Heterozygous c. 79 G>A, c. 341 A>G |
| S14 | Heterozygous GJB2 gene c. 299_300 del AT | GJB2 | Wild type |
| S15 | Heterozygous GJB2 gene c. 235 del C | GJB2 | Wild type |
| S16 | Heterozygous GJB2 gene c. 235 del C | GJB2 | Heterozygous c. 79 G>A |
| S17 | Heterozygous GJB2 gene c. 235 del C | GJB2 | Heterozygous c. 79 G>A, c. 341 A>G |
| S18 | Heterozygous GJB2 gene c. 235 del C | GJB2 | Wild type |
| S19 | Heterozygous GJB3 gene c. 538 C>T | GJB2, GJB3 | Wild type |
| S20 | Heterozygous GJB2 gene c. 235 del C | GJB2 | Heterozygous c. 79 G>A, c. 341 A>G |
| S21 | Heterozygous GJB2 gene c. 235 del C | GJB2 | Wild type |
| S22 | Heterozygous GJB2 gene c. 299_300 del AT | GJB2 | Heterozygous c. 79 G>A |
| S23 | Heterozygous GJB2 gene c. 235 del C | GJB2 | Wild type |
| S24 | Heterozygous GJB2 gene c. 235 del C | GJB2 | Heterozygous c. 79 G>A, c. 341 A>G |
| S25 | Heterozygous GJB2 gene c. 235 del C | GJB2 | Wild type |
| S26 | Heterozygous SLC26A4 gene c. 919–2 A>G | SLC26A4 | Wild type |
| S27 | Heterozygous GJB2 gene c. 235 del C | GJB2 | Wild type |
| S28 | Heterozygous SLC26A4 gene c. 919–2 A>G | SLC26A4 | Heterozygous IVS 7-18 T>G, IVS 11+47 T>C |
| S29 | Heterozygous SLC26A4 gene c. 919–2 A>G | SLC26A4 | Wild type |
| S30 | Heterozygous SLC26A4 gene c. 919–2 A>G | SLC26A4 | Wild type |
| S31 | Heterozygous GJB2 gene c. 235 del C | GJB2 | Wild type |
| S32 | Heterozygous c. 176_191 del 16 bp | GJB2 | Wild type |
| S33 | Heterozygous GJB2 gene c. 235 del C | GJB2 | Heterozygous c. 79 G>A, c. 341 A>G |
| S34 | Heterozygous GJB2 gene c. 235 del C | GJB2 | Wild type |
| S35 | Heterozygous GJB2 gene c. 235 del C | GJB2 | Wild type |
| S36 | Heterozygous SLC26A4 gene c. 919–2 A>G | SLC26A4 | Wild type |
| S37 | Heterozygous SLC26A4 gene c. 919–2 A>G | SLC26A4 | Heterozygous IVS 11+47 T>C |
| S38 | Heterozygous c. 176_191 del 16 bp | GJB2 | Heterozygous c. 608 T>C |
| S39 | Heterozygous SLC26A4 gene c. 2168 A>G | SLC26A4 | Wild type |
| S40 | Heterozygous GJB2 gene c. 235 del C | GJB2 | Wild type |
| S41 | Heterozygous GJB2 gene c. 235 del C | GJB2 | Heterozygous c. 79 G>A, c. 341 A>G |
| S42 | Heterozygous GJB2 gene c. 235 del C | GJB2 | Wild type |
| S43 | Heterozygous GJB2 gene c. 299_300 del AT | GJB2 | Wild type |
| S44 | Heterozygous GJB2 gene c. 235 del C | GJB2 | Wild type |
| S45 | Heterozygous SLC26A4 gene c. 2168 A>G | SLC26A4 | Wild type |
| S46 | Heterozygous GJB2 gene c. 235 del C | GJB2 | Wild type |
| S47 | Heterozygous GJB2 gene c. 235 del C | GJB2 | Heterozygous c. 79 G>A, c. 341 A>G |
| S48 | Heterozygous GJB2 gene c. 235 del C | GJB2 | Heterozygous c. 79 G>A, c. 341 A>G |
| S49 | Heterozygous GJB2 gene c. 235 del C | GJB2 | Heterozygous c. 79 G>A, c. 341 A>G |
| S50 | Heterozygous SLC26A4 gene c. 919–2 A>G | SLC26A4 | Wild type |
| S51 | Heterozygous GJB2 gene c. 235 del C | GJB2 | Heterozygous c. 79 G>A, c. 341 A>G |
| S52 | Heterozygous GJB2 gene c. 235 del C | GJB2 | Heterozygous c. 79 G>A, c. 341 A>G |
| S53 | Heterozygous GJB2 gene c. 235 del C | GJB2 | Heterozygous c. 79 G>A, c. 109 G>A, c. 341 A>G |
| S54 | Heterozygous GJB2 gene c. 299_300 del AT | GJB2 | Heterozygous c. 79 G>A, c. 341 A>G |
| S55 | Heterozygous SLC26A4 gene c. 919–2 A>G | SLC26A4 | Wild type |
| S56 | Heterozygous SLC26A4 gene c. 919–2 A>G | SLC26A4 | Wild type |
| S57 | Heterozygous GJB2 gene c. 235 del C | GJB2 | Heterozygous c. 608 T>C |
| S58 | Heterozygous GJB2 gene c. 235 del C | GJB2 | Wild type |
| S59 | Heterozygous SLC26A4 gene c. 919–2 A>G | SLC26A4 | Wild type |
| S60 | Heterozygous SLC26A4 gene c. 919–2 A>G | SLC26A4 | Wild type |
| S61 | Heterozygous GJB2 gene c. 235 del C | GJB2 | Wild type |
| S62 | Heterozygous GJB2 gene c. 235 del C | GJB2 | Heterozygous c. 608 T>C |
| S63 | Heterozygous GJB2 gene c. 299_300 del AT | GJB2 | Wild type |
| S64 | Heterozygous GJB2 gene c. 299_300 del AT | GJB2 | Heterozygous c. 79 G>A, c. 341 A>G |
| S65 | Heterozygous GJB2 gene c. 235 del C | GJB2 | Heterozygous c. 79 G>A, c. 341 A>G, c. 608 T>C |
| S66 | Heterozygous GJB2 gene c. 299_300 del AT | GJB2 | Heterozygous c. 235 del C |
| S67 | Heterozygous GJB2 gene c. 235 del C | GJB2 | Wild type |
| S68 | Heterozygous GJB2 gene c. 235 del C | GJB2 | Heterozygous c. 608 T>C |
| S69 | Heterozygous SLC26A4 gene c. 919–2 A>G | SLC26A4 | Wild type |
| S70 | Heterozygous SLC26A4 gene c. 919–2 A>G | SLC26A4 | Wild type |
| S71 | Heterozygous SLC26A4 gene c. 919–2 A>G | SLC26A4 | Wild type |
| S72 | Heterozygous GJB2 gene c. 299_300 del AT | GJB2 | Heterozygous c. 79 G>A |
| S73 | Heterozygous GJB2 gene c. 235 del C | GJB2 | Homozygous c. 79 G>A |
| S74 | Heterozygous GJB3 gene c. 538 C>T | GJB2, GJB3 | Heterozygous c. 79 G>A, c. 341 A>G |
| S75 | Heterozygous GJB2 gene c. 235 del C | GJB2 | Wild type |
| S76 | Heterozygous GJB2 gene c. 235 del C | GJB2 | Wild type |
| S77 | Heterozygous GJB2 gene c. 235 del C | GJB2 | Heterozygous c. 79 G>A, c. 341 A>G |
| S78 | Heterozygous GJB2 gene c. 235 del C | GJB2 | Heterozygous c. 79 G>A, c. 235 del C, c. 341 A>G |
| S79 | Heterozygous SLC26A4 gene c. 919–2 A>G | SLC26A4 | Wild type |
| S80 | Heterozygous GJB2 gene c. 235 del C | GJB2 | Heterozygous c. 79 G>A, c. 341 A>G |
| S81 | Heterozygous GJB2 gene c. 299_300 del AT | GJB2 | Heterozygous GJB2 gene c. 235 del C |
| S82 | Heterozygous GJB2 gene c. 235 del C | GJB2 | Heterozygous GJB2 gene c. 235 del C |
| S83 | Heterozygous SLC26A4 gene c. 919–2 A>G | SLC26A4 | Heterozygous IVS 11+47 T>C |
| S84 | Heterozygous SLC26A4 gene c. 919–2 A>G | SLC26A4 | Wild type |
| S85 | Heterozygous GJB2 gene c. 235 del C | GJB2 | Wild type |
| S86 | Heterozygous GJB2 gene c. 235 del C | GJB2 | Heterozygous c. 299_300 del AT |
| S87 | Heterozygous SLC26A4 gene c. 919–2 A>G | SLC26A4 | Heterozygous c.1548 ins C, IVS11+47 T>C |
| S88 | Heterozygous GJB2 gene c. 235 del C | GJB2 | Wild type |
| S89 | Heterozygous SLC26A4 gene c. 919–2 A>G | SLC26A4 | Wild type |
| S90 | Heterozygous GJB2 gene c. 235 del C | GJB2 | Heterozygous c. 79 G>A, c. 512 ins AACG, c. 341 A>G |
| S91 | Heterozygous GJB2 gene c. 235 del C | GJB2 | Heterozygous c. 109 G>A |
| S92 | Heterozygous GJB2 gene c. 235 del C | GJB2 | Wild type |
| S93 | Heterozygous SLC26A4 gene c. 919–2 A>G | SLC26A4 | Wild type |
| S94 | Heterozygous GJB2 gene c. 235 del C | GJB2 | Wild type |
| S95 | Heterozygous GJB2 gene c. 299_300 del AT | GJB2 | Wild type |
